# I_2_-Catalyzed Oxidative Condensation of Aldoses with Diamines: Synthesis of Aldo-Naphthimidazoles for Carbohydrate Analysis

**DOI:** 10.3390/molecules15031340

**Published:** 2010-03-05

**Authors:** Chunchi Lin, Wei-Ting Hung, Chien-Yuan Kuo, Kuo-Shiang Liao, Yin-Chen Liu, Wen-Bin Yang

**Affiliations:** Genomics Research Center, Academia Sinica, No. 128, Academia Road Section 2, Nankang, Taipei 11529, Taiwan

**Keywords:** iodine, saccharides, aldo-imidazole (aldo-IM), matrix-assisted laser desorption/ionization time-of-flight mass spectrometry (MALDI–TOF MS)

## Abstract

A novel method for the conversion of unprotected and unmodified aldoses to aldo-imidazoles has been developed. Using iodine as a catalyst in acetic acid solution, a series of mono- and oligosaccharides, including those containing carboxyl and acetamido groups, undergo an oxidative condensation reaction with aromatic vicinal diamines at room temperature to give the corresponding aldo-imidazole products in high yields. No cleavage of the glycosidic bond occurs under the mild reaction conditions. The compositional analysis of saccharides is commonly realized by capillary electropheresis of the corresponding aldo-imidazole derivatives, which are easily synthesized by the reported iodine-promoted oxidative condensation. In addition, a series of aldo-imidazoles were determined by matrix-assisted laser desorption/ionization time-of-flight mass spectrometry (MALDI–TOF MS) to analyze molecular weight and ion intensity. The diamine-labeled saccharides showed enhanced signals in MALDI–TOF MS. The combined use of aldo-imidazole derivatization and mass spectrometric analysis thus provides a rapid method for identification of saccharides, even when less than 1 pmol of saccharide is present in the sample. These results can be further applied to facilitate the isolation and analysis of novel saccharides.

## Introduction

Molecular iodine has been used in organic synthesis for a long time. In recent years this reagent has received considerable attention as an inexpensive, nontoxic, readily available catalyst for various organic transformations under mild and convenient conditions and affording the corresponding products in excellent yields with high selectivity [1,2,3,4,5,6,7,8]. The usage of iodine in organic transformations ranges from stoichiometric levels to catalytic amounts. Our ongoing interest is in the iodine catalyzed condensation reaction of various aldoses with aromatic vicinal diamines to form the corresponding saccharide-aldo-imidazoles (IMs) for carbohydrate labeling and determination [9].

**Scheme 1:**
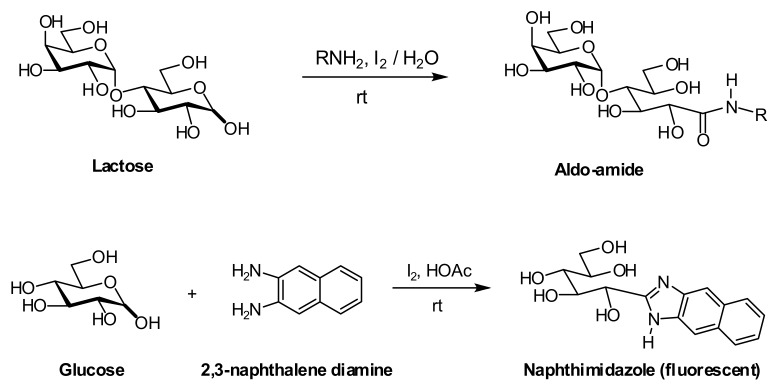
Use of molecular iodine for aldose oxidation [9,12,13].

Carbohydrates are essential materials in many biological processes. They can maintain and modulate the function of proteins [10]. Many conjugation methods have been developed by anchoring carbohydrates to solid supports and others [11]. However, some of these methods may be time-consuming and result in low overall yield due to the many preparation steps needed. Direct conjugation of amines or diamines with aldehydes on the reducing end of an aldose by forming an amide or an imidazole group has been developed [9,12,13,14,15]. By using molecular iodine, an aldose was directly coupled with an amine through an amide bond in aqueous solution. Aldoses undergo an oxidative amidation with a variety of aliphatic amines, bifunctional amines, α-amino esters, and peptides. Lin *et al*. reported a conversion of unmodified aldoses to aldo-naphthimidazoles using iodine and aromatic *ortho*-diamine in acetic acid solution [9]. A series of mono- and oligosaccharides, including those containing carboxyl and acetamido groups, undergo an oxidative condensation reaction with 2,3-naphthalenediamine at room temperature to give the corresponding aldo-naphthimidazole products (Scheme 1).

Carbohydrates cannot be easily analyzed due to the isolation and detection difficulties. Because carbohydrate molecules lack an intrinsic chromophore, suitably labeled saccharides are desirable for their compositional analysis. For example, conversion of aldoses by reductive amination [16] at the reducing terminus has been a common practice to give the derivatives suitable for UV, fluorescence and mass analyses. In continuation of our studies of novel transformations of aldoses using diamines and iodine [9], we describe herein an improved protocol for the synthesis of various aldo-imidazoles and analogues by direct oxidative condensation of aldoses, including mono-, di-, and oligosaccharides, with aromatic vicinal diamines in the presence of iodine. We also demonstrate that the compositional analysis of carbohydrate molecules is facilitated by incorporating the imidazole moiety as a UV or fluorescent label for sensitive detection. The compositional analysis of saccharides is realized by the CE and HPLC analysis of the resulting aldo-imidazole derivatives.

Matrix-assisted laser desorption/ionization time-of-flight mass (MALDI–TOF MS) has been successfully developed as a soft ionization method for glycosides and glycoconjugates [17,18]. In recent years, many mass spectrometric studies have focused on the analysis of biological oligo-saccharides [19,20]. Direct detection of saccharides in sulfuric acid/phenol has been commonly used, but it cannot derive information on molecular weights of different saccharides or distinguish among pentoses, hexoses and unusual sugars. On the other hand, MALDI–MS is a practical technique for more qualitative and mass determinations. In our previous study [21,22], we found that non-derivative and derivative polysaccharides were successfully analyzed by straightforward MALDI mass spectrometry when 2’,4’,6’-trihydroxyacetophenone (THAP) or 2,5-dihydroxybenzoic acid (2,5-DHB) was used as matrix. Perreault *et al*. have recently demonstrated the application of 2,5-DHB/aniline and 2,5-DHB/*N,N*-dimethylaniline as matrices for automated identification and quantitative analysis of native oligosaccharides by MALDI–MS [23]. However, there still exist some challenges in the analysis of saccharides due to the inherently low ionization efficiency of saccharides. Therefore saccharides are often derivatized before MALDI–MS to remedy this problem. It is well known that increasing the hydrophobicity of glycans improves the mass spectrometric ionization response. To improve the sensitivity and the detectable mass range of saccharides, several derivatization methods have been developed, such as peracetylation, permethylation, reductive amination, and oxime formation. The sensitivity of saccharide derivatives showed enhancements over that of the free oligosaccharides in MALDI–MS spectrometry. Nonetheless, the reductive amination still has limitation on modification of saccharides. In addition, the high content of salts in the products needs to be removed to increase the signal level with mass spectrometric analysis. We demonstrate herein a promising method for saccharide derivativation via the iodine catalyzed oxidative condensation of aldoses with aromatic diamines, which are efficiently prepared in high yields even with little sample. For example, saccharides were successfully converted to the aldo-IM derivatives that showed high sensitivity for MALDI–MS analysis. This is the first study to compare the mass signals of saccharide and its imidazole derivatives in saccharide determination.

## Results and Discussion

**Figure 1:**
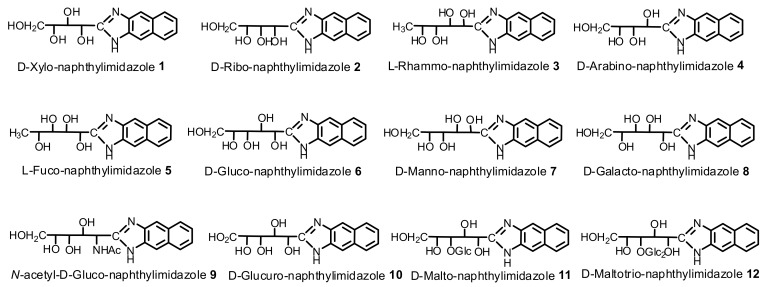
Structures of aldo-naphthimidazole compounds used for monosaccharide compositional analysis by capillary electropheresis.

Molecular iodine is a convenient and environmentally benign oxidizing agent in organic synthesis. Two recent studies show that aliphatic and aromatic aldehydes react with 1,2-diamines to give the corresponding imidazolines in the presence of stoichiometric amounts of iodine [8,12]. The reactions are conducted at high temperature in protic solvents (H_2_O or *t*-BuOH) using K_2_CO_3_ as the base to neutralize the hydroiodic acid generated. So far, these methods have been applied less to aldoses, presumably because the hydroxyl groups in aldoses may also be oxidized by I_2_/K_2_CO_3_ at high temperature. Our goal in this study was to improve the reaction conditions by using catalytic amounts of iodine and HOAc as solvent to facilitate formation of the Schiff base and product. We tested the direct oxidative condensation of D-glucose with 2,3-naphthalenediamine in the I_2_/HOAc system at an ambient temperature for 6~12 h. We also surveyed the oxidative condensation reaction of various aldoses with 2,3-naphthalene diamines under the same conditions. Thus, aldoses including xylose, ribose, rhamnose, arabinose, fucose, glucose, mannose, galactose, *N*-acetylglucosamine, glucuronic acid and some oligosaccharides reacted with 2,3-naphthalenediamine to yield its NAIM derivatives **1**-**12** (Figure 1). The oxidative condensation of saccharides with 2,3-napthalenediamine occurred readily at room temperature without using KI or K_2_CO_3_ additives. Thus, D-glucose was treated with 2,3-naphthalenediamine (1.1 equiv) and catalytic amount iodine (0.1 equiv) in acetic acid at room temperature for 6 hours to give D-gluconaphthimidazole **6** in 98% yield by a EtOAC trituration or flash column chromatography. The formation of the products were monitored by TLC analysis and their structures determined from their NMR spectra. For example, the ^13^C-NMR spectrum of **6** in DMSO-*d*6 solution showed a signal at *δ* 156.2 attributable to the C-2 of the newly formed naphthimidazole ring. In the ^1^H-NMR spectrum (DMSO-*d*6), the naphthyl protons in naphthimidazole appeared at lower fields at *δ* 7.71 and 7.48. The proton at the C-1’ position of **6** occurred at *δ* 5.16 as a doublet with a small coupling constant (5.2 Hz). The similar derivatives of neutral saccharides compound **1-8**, amino-saccharide **9**, acidic saccharide **10**, and oligo-saccharides **11, 12** were also synthesized in good yield [9]. 

**Scheme 2:**
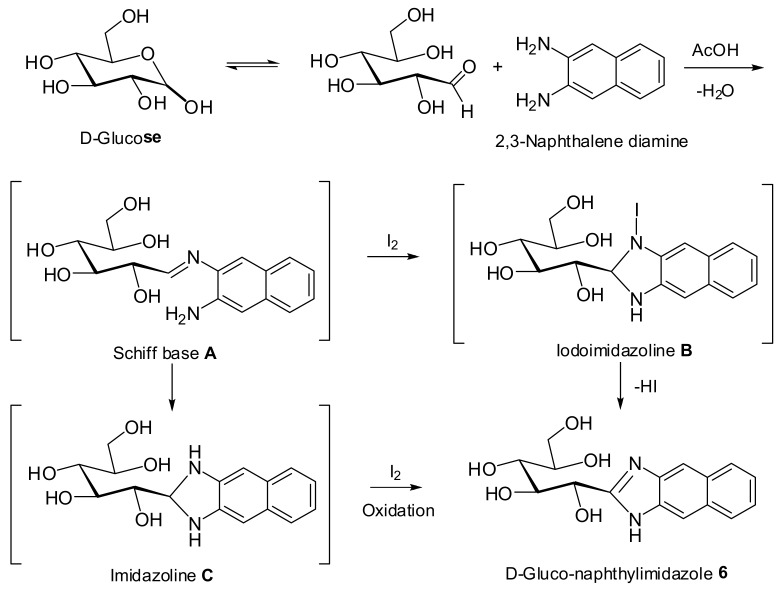
The proposed mechanism for synthesis of aldo-naphthylimidazoles by used molecular iodine as the oxidizing agent.

We propose a speculative mechanism for this iodine-catalyzed oxidative condensation reaction. The reaction was initiated by formation of a Schiff base **A** by condensation of the aldose with one of the NH_2_ group in 2,3-naphthalene diamine (Scheme 2). Here, acetic acid is an appropriate solvent for the transformation of aldoses (hemiacetals form) to an open ring structure (aldehyde form) to facilitate the formation of Schiff base **A**. The subsequent nucleophilic addition of the other amino group to the Schiff base would give an intermediate imidazoline **C** and then **C** would be oxidized by iodine to afford the desired aldo-naphthylimidazole product. The product might also be obtained via a different pathway from Schiff base structure **A**. *N*-iodination of Schiff base **A** could facilitate the formation of intermediate iodoimidazoline **B**, and the desired product would be obtained by the subsequent elimination of an HI molecule. In the HOAC media, the released I^–^ ions might be oxidized in air to regenerate iodine molecule in this catalytic system. This is a first example of using iodine as an oxidative agent in saccharide labeling by aldo-naphthylimidazole formation.

**Figure 2:**
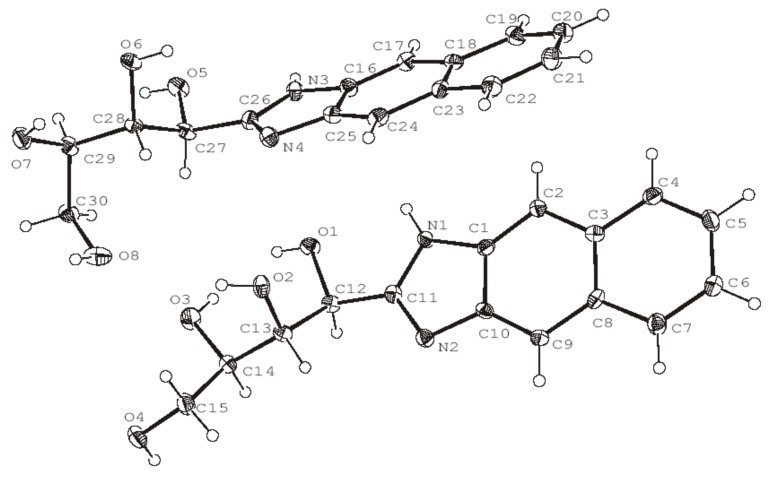
X-ray structure of the xylo-naphthimidazole.

The molecular structure of compound **1** was determined by X-ray diffraction (Figure 2). Based on the structure, the naphthylimidazole group has a planar geometric shape due to its aromatic resonance nature. Since the naphthylimidazole group might make this NAIM derivative more rigid, it can slow down the free rotation of the aldose on C11-C12 than in those amine derivatives with aldose C1-C2 bonds. Due to this geometric property of NAIM derivatives we studied the chromatography of these NAIM derivatives for application in saccharide compositional analysis.

**Figure 3:**
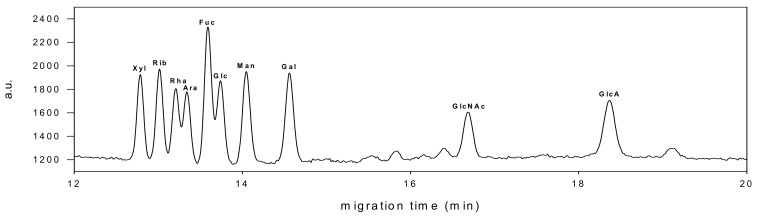
Capillary electrophoresis chromatograph of pento- and hexo-naphthimidazoles on uncoated fused-silica capillary (50/60.2 cm × 50 μm) at 30 °C. The mobile phase is Tris buffer (500 mM, pH 2.5) at a flow rate of 0.8 mL/min. The UV detection wavelength is 326 nm. The sample is a mixture of aldonaphthimidazoles, including those derived from D-xylose (**1**, peak 1), D-ribose (**2**, peak 2), L-rhammose (**3**, peak 3), D-arabinose (**4**, peak 4), L-fucose (**5**, peak 5), D-glucose (**6**, peak 6), D-mannose (**7**, peak 7), D-galactose (**8**, peak 8), *N*-acetyl-D-glucosamine (**9**, peak 9) and D-glucuronic acid (**10**, peak 10) at a concentration of 10 ppm for each component.

Our preliminary study indicated that several aldo-naphthimidazoles derived from pentoses and hexoses were resolved by HPLC on reversed-phase columns [9]. The analysis of aldonaphthimidazoles can be further investigated by using capillary electrophoresis (CE) as a high-resolution and sensitive method (Figure 3). Here, we have monitored aldonaphthimidazoles **1**-**10**. Not only neutral saccharides but also acetamido group containing saccharide **9** and carboxyl group containing saccharide **10** are available for capillary electrophoresis chromatography. The detection limit was around 1 ppm. This is a good and fast methodology for sugar compositional analysis with minor sample loading. Though fluorescent labels can be introduced into aldoses by reductive amination [16], this method still has limitations on modification of oligosaccharides. Our current method using the iodine-promoted oxidative condensation reactions provides an alternative way to convert aldoses into highly fluorescent aldo-naphthimidazoles in a direct and efficient manner.

**Figure 4:**
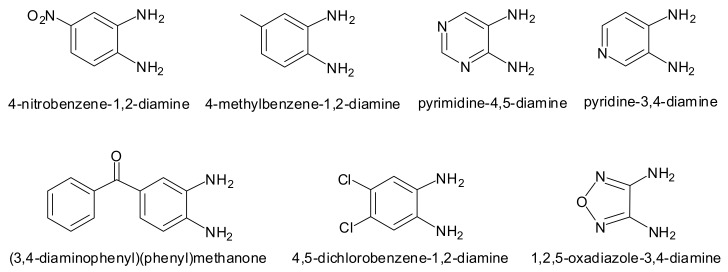
Various aromatic diamines were used in the iodine-promoted oxidative condensation reactions with aldose.

A recent publication by Perreault *et al*. has demonstrated the application of 2,5-DHB/aniline and 2,5-DHB/*N,N*-dimethylaniline matrices for automated identification and quantitative analysis of native oligosaccharides by MALDI–MS [23]. However, on-target formation of aniline Schiff base derivatives shows a lower intensity than native glycans. It is presumably caused by incomplete formation of the unstable Schiff base that may be hydrolyzed during the MALDI–MS process. Therefore, it is better to use stable derivatives for investigation of saccharides’ ionization efficiencies and sensitivity in MALDI–MS determination. Our study found that aldo-NAIM derivatives are able to enhance the sensitivity of saccharides in mass spectrometry detection compared with the corresponding underivatized saccharides [24]. We thus synthesized some aldo-imizazole derivatives with aromatic vicinal diamines to see if they could enhance the saccharide signal in MALDI–MS determinations. The similar reactions of D-glucose and D-maltotriose with various aromatic diamines (Figure 4) and a catalytic amount iodine in aqueous AcOH solution, gave the expected aldo-imidazole compounds in good yields. The iodine-promoted oxidative condensation reactions of D-glucose and D-maltotriose with 4,5-dichloro-1,2-benzenediamine and 4-methyl-1,2-benzenediamine were also carried out effectively at room temperature to afford the aldo-imidazoles **13, 14** and **15, 16**, whereas the reactions with 4-nitro-1,2-benzenediamine and (3,4-diaminophenyl) (phenyl)methanone to give aldo-imidazole derivatives **17**-**20** were conducted at an elevated temperature during to their low reactivity. It appeared that the oxidative condensation reactions were accelerated by the electron-donating substituents and decelerated by the electron-withdrawing groups on the phenyl ring of *ortho*-phenylenediamine. 

As known, peptides and some heteroatom compounds are more sensitive than saccharides in mass determination due to their facility for ionization [17,18]. To apply it to the advanced study of saccharides by mass determination we also used this catalytic iodine reaction to condense D-malto-triose with pyrimidine-4,5-diamine, pyridine-3,4-diamine, and 1,2,5-oxadiazole-3,4-diamine to give the corresponding maltotrio-imidazole derivatives **21**, **22**, and **23**. The increase of the number of hetero-atoms in the aldo-imidazoles was in order to investigate if it is possible to enhance the sensitivity of the saccharide signals for mass spectrometry detection. Aromatic *ortho*-diamine groups were also used instead of aliphatic α-diamines, α-hydroxylamines, and α-thiolamines for this reaction. However, this operation failed to give the desired aldo-imidazole, aldo-thiazole and oxazole products. The low reactivity might due to their geometry and difficulty to form an aromatic ring through this iodine-promoted oxidative condensation reaction. We also surveyed the oxidative condensation reaction of D-glucose with 2,3-naphthalenediamine and iodine in various solvents to investigate the use of solvents other than acetic acid, including CH_3_CN, DMF, DMSO, MeOH, and H_2_O. None of them are suitable media for the desired reaction.

**Figure 5:**
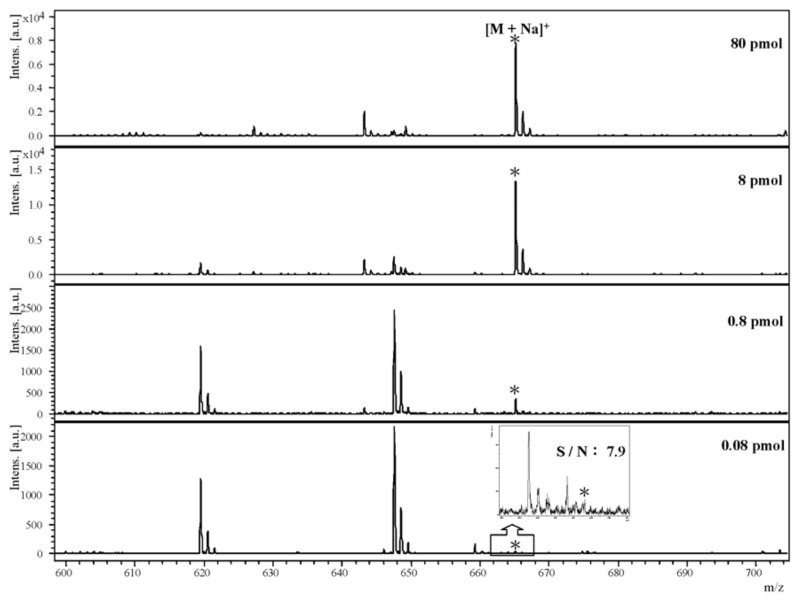
MALDI–TOF MS of maltotrio–naphthimidazole with loading of 80, 8, 0.8 and 0.08 pmol, respectively, using 50 nmol of 2,5-DHB as a matrix. The asterisk (＊) denotes the [Glc_3_–NAIM + Na]^+^ ion at *m/z* 665.2. The signals of matrix become obvious at low analyte concentration.

The molecular weight of aldo-imidazole derivatives from iodine-promoted oxidative condensation reactions were determined by MALDI–TOF MS to investigate if it is possible to enhance the sensitivity of the saccharide signals in mass spectrometry detection. Our current method for a direct conversion of maltotriose to the corresponding maltotrio-naphthylimidazole product is applicable to the oxidative condensation of saccharides with various aromatic *ortho*-diamines. We have succeeded in preparing aldo-imidazole derivatives. These imidazole labeled saccharides are desirable for MS analysis. In general, the efficiency of using MALDI for saccharide detection is significantly lower than that for proteins. Therefore, it is critical to increase the ionization efficiency for saccharides. One approach to achieve this is via chemical modification of the saccharide samples. In our synthetic study, we provided an efficient method for the conversion of various saccharides to the corresponding imidazole derivatives, which showed the sodium adduct ions [M + Na]^+^ with significant enhancement in the MALDI–MS spectra using 2,5-DHB or THAP as the matrix [24]. For example, the molecular iodine catalytic oxidative condensation of maltotrio-naphthylimidazole **12** was determined by MALDI–TOF mass at the nmol-scale, and an aliquot (0.08–800 pmol) of the saccharide–NAIM derivative (Figure 5). 

No cleavage of the glycosidic bond occurred under these reaction conditions. In comparison with the underivatized saccharides the iodine-promoted NAIM product shows a mass sensitivity advantage in the MALDI–MS spectra. The saccharide–NAIM derivatives are stable and co-crystallize well easier with the matrix than underivatized saccharides. The naphthimidazole derivatized maltotriose showed the [Glc_3_–NAIM + Na]^+^ ion at *m/z* 665.2, marked as *, at 80 pmol, 8 pmol, 0.8 pmol and 0.08 pmol in order, and with a better intensity (ca. 10 times high) than the [Glc_3_ + Na]^+^ ion of native maltotriose at *m/z* 527.2 (data not shown). The NAIM derivative **12** not only has a higher mass signal but also it has less interference with the matrix signals in the mass spectrogram. THAP and a combination of 2,5-DHB/*N,N*-dimethylaniline as the matrix have been shown to be an applicable matrix in MALDI–MS analysis for saccharides [21,23]. However, the NAIM derivatived maltotriose still showed a higher [Glc_3_–NAIM + Na]^+^ signal in 2,5-DHB. We thus used 2,5-DHB as the matrix throughout this MALDI–MS study.

**Figure 6:**
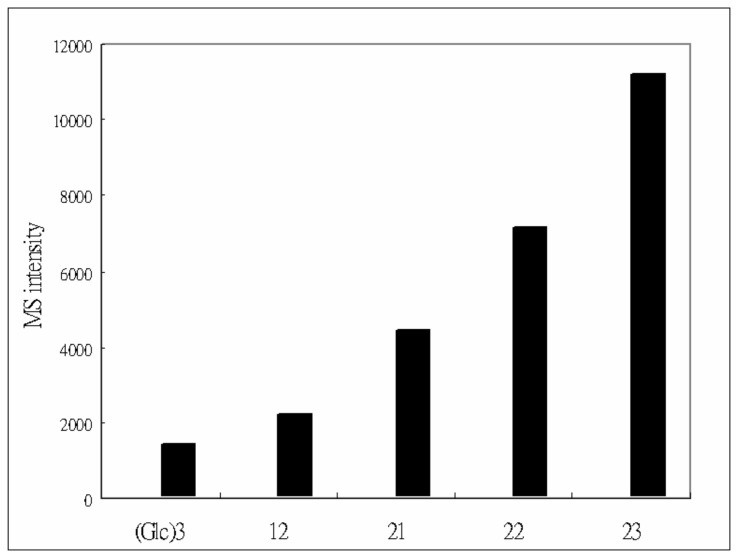
The comparison of mass indensity in MALDI-TOF MS with 2,5-DHB as matrix. The signal sensitivity of maltotriose (Glc)_3_, maltotrio-NAIM **12** and heteroatom derivatived maltotrio-imidazoles **21**, **22**, and **23** are shown. An 8 pmol of sample was deposited per spot. Spectra were obtained by accumulating 1000 laser shots. The matrix concentration was set at 50 nmol.

In practice, conversion of maltotriose (Glc_3_) to its naphthimidazole derivative was achieved on nmol ~ pmol scales, and the product was diluted and directly subjected to MALDI–MS analysis and the signals can still be detected using a general protocol. The signal corresponding to the sodium adduct of Glc_3_–NAIM was readily detected at *m/z* 665.2 Da. Even with a low sample content of 0.08 pmol as well as a 1,000 times higher ratio of 2,5-DHB/aldo–NAIM, the [aldo–NAIM + Na]^+^ ion peak was still obvious without interference from matrix signals. Since each matrix load was fixed at 50 nmol, the major signals of matrix at *m/z* 619.5 and 647.5 become obvious at low analyte concentration. In general, the higher matrix-to-analyte ratio gave the stronger analyte signals. The desorption/ionization efficiency of maltotriose, maltotrio–NAIM **12** and other maltotrio-imidazoles **21**-**23** which have heteroatoms in the aromatic ring, such as pyrimidine-4,5-diamine, pyridine-3,4-diamine and 1,2,5-oxadiazole-3,4-diamine were compared. The MALDI–MS of a mixture containing equal amounts of maltotriose and maltotrio-imidazoles (32 pmol each) showed the sodium adduct ions at *m/z* 527.1 [maltotriose + Na]^+^, 665.2 [**12** + Na]^+^, 619.2 [**21** + Na]^+^, 618.2 [**22** + Na]^+^, and 609.2 [**23** + Na]^+^, respectively. The corresponding mass intensities of maltotriose series derivatives are shown in Figure 6. The Glc_3_–imidazole derivatives **21**-**23** displayed a significantly higher sensitivity compared to a reference sample of maltotrio-NAIM **12** and underivatived maltotriose. 

Among 1,2,5-oxadiazole-3,4-diamine derivatived compound **23** showed the best sensitivity signal in MALDI–TOF MS. In this study, all of the aromatic *ortho*-diamine derivatived saccharide–imidazoles displayed a significantly higher sensitivity compared to unmodified saccharides. The iodine catalytic saccharide condensation reaction thus provided an application for enhancement of saccharide sensitivity in MALDI–TOF MS detection. 

## Experimental

### Materials

Aromatic diamines and solvents (ethyl acetate, methanol, dimethyl sulfoxide, and acetic acid) used in this labeling reaction were purchased from Merck & Co., Inc. Mono- and oligosaccharides and the matrices of 2’,4’,6’-trihydroxyacetophenone (THAP) and 2,5-dihydroxybenzoic acid (2,5-DHB) were obtained from Sigma Chem. Co. All chemicals were analytical grade and used without further purification and double distilled water or buffer solution was used throughout for aldo-imidazole purification. 

### Instrumentation

The MALDI–TOF mass spectrometer which was used to acquire the spectra was a Voyager Elite (Applied Biosystems, Foster City, CA. USA), equipped with a nitrogen pulsed laser (337 nm). The accelerating voltage was set at 20 kV in either a positive or negative ion mode. Spectra from 800-1,000 laser shots were accumulated to obtain the final spectrum. Laser energy per pulse was calibrated with a laser power meter (PEM 101, Germany). The delay extraction time could be adjusted from 10 ns to 500 ns. The grid voltage was set up at 95% of the accelerating voltage. The laser beam diameter was measured as ~100 μm on the sample target. The laser fluence applied was in the range of 50~300 mj/cm^2^ and the vacuum inside the flight tube was always kept between 10^-7^–10^-6^ Torr. Capillary electrophoresis (Backman Coulter/USA, P/ACE MDQ) with a uncoated fused-silica capillary column (50/60.2 cm × 50 μm). The UV detection wavelength is set at 326 nm. High-performance anion-exchange chromatography with a pulsed amperometric detection (HPAEC–PAD) for sugar composition analysis was performed on a Dionex BioLC system (Dionex, Sunnyvale, CA, USA) using a CarboPac PA10 analytical column (2 mm × 250 mm) and a CarboPac PA10 guard column (2 mm × 50 mm). The NMR spectra were recorded on Bruker 600 MHz NMR spectrometer (Bruker BioSpin GmbH, Rheinstetten, Germany) with a 5 mm Cryoprobe DCI ^1^H/^13^C.

### Preparation of saccharide-imidazole derivatives

**Figure 7:**
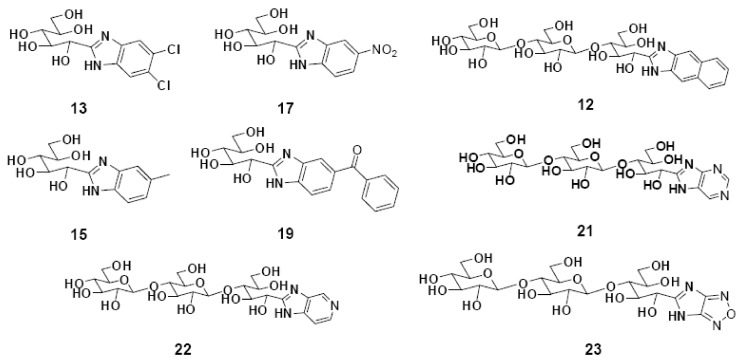
The compound list of synthesized aldo–IM derivatives.

Typically, a mixture of glucose (3.6 mg, 0.020 mmol), 2,3-naphthalenediamine (3.5 mg, 0.022 mmol), and iodine (0.5 mg, 0.002 mmol) in AcOH (5.0 mL) was stirred at room temperature in open air. The reaction was completed in 6~12 hours as indicated by the TLC analysis. The reaction mixture was triturated with EtOAc to give precipitates which were collected by filtration. The gluco-naphthimidazole products prepared as such was practically pure enough for characterization. This protocol is also applicable to the reaction of various aldoses (mono-, and oligo-saccharides) to react with various aromatic diamines (Figure 7). When the amounts of sample is low (nano- or pico-mole), we used the corresponding reagents in a diluted solution for saccharide labeling. The saccharide–NAIM product was directly determined by MALDI–MS and CE analysis without further purification.

*(1’S,2’R,3’R,4’R)-1H-2-[(1,2,3,4,5-Pentahydroxy)pentyl]-3,4-dichlorobenzimidazole* (C_12_H_14_Cl_2_N_2_O_5_, **13**): brown solid; mp = 101–103 ^o^C; ^1^H-NMR (DMSO-*d_6_*, 600 MHz) δ 7.85 (2 H, s), 5.06 (1 H, s), 4.14 (1 H, s), 3.53 (1 H, s), 3.49 (2 H, s), 3.36 (1 H, s); ^13^C-NMR (DMSO-*d_6_*, 150 MHz) δ 158.9 (2 ×), 133.8 (2 ×), 126.2 (2 ×), 115.7, 71.5, 71.0, 69.8, 68.4, 63.3- HRMS (ESI) calcd. for C_12_H_15_Cl_2_N_2_O_5_: 337.0358; found: *m/z* 337.0353 (M^+^ + H).

*(1’S,2’R,3’R,4’R)-1-H-2-[(1,2,3,4,5-Pentahydroxy)pentyl]-4-methylbenzimidazole* (**15**): brown solid; mp = 81–82 °C; ^1^H-NMR (DMSO-*d_6_*, 600 MHz) *δ* 7.54 (1 H, d, *J* = 8.3 Hz ), 7.44 (1 H, s), 7.23 (1 H, d, *J* = 8.3 Hz), 5.10 (1 H, d, *J* = 5.2 Hz), 4.16 (1 H, d, *J* = 4.6 Hz), 3.55 (3 H, m), 3.37 (1 H, dd, *J* = 10.4, 4.6 Hz), 2.43 (3 H, s); ^13^C-NMR (DMSO-*d_6_*, 150 MHz) *δ* 155.3, 134.2, 132.6, 130.6, 126.0, 113.8, 113.6, 71.4, 71.1, 69.6, 68.0, 63.3; HRMS (ESI) calcd. for C_13_H_19_N_2_O_5_: 283.1294; found: *m/z* 283.1288 (M^+^ + H).

*(1’S,2’R,3’R,4’R)-1-H-2-[(1,2,3,4,5-Pentahydroxy)pentyl]-4-nitrobenzimidazole* (**17**): brown solid; mp = 169–170 °C; ^1^H-NMR (DMSO-*d_6_*, 600 MHz) *δ* 8.40 (1 H, s), 8.11 (1 H, s), 7.71 (1 H, d, *J* = 5.8 Hz), 5.06 (1 H, s), 4.12 (1 H, s), 3.57 (4 H, br); ^13^C-NMR (DMSO-*d_6_*, 150 MHz) *δ* 161.3, 142.8, 141.2, 137.0, 118.1, 114.8, 111.6, 71.9, 71.3, 70.7, 69.6, 63.4; HRMS (ESI) calcd. for C_12_H_16_N_3_O_7_: 314.0988; found: *m/z* 314.0983 (M^+^ + H).

*(1’S,2’R,3’R,4’R)-1-H-2-[(1,2,3,4,5-Pentahydroxy)pentyl]-4-benzoxylbenzimidazole* (**19**): brown solid; mp = 143–144 °C; ^1^H-NMR (DMSO-*d_6_*, 600 MHz) *δ* 7.95 (1 H, s ), 7.74 (4 H, s), 7.69 (1 H, br), 7.59 (2 H, br), 5.10 (1 H, d, *J* = 5.1 Hz), 4.17 (1 H, d, *J* = 4.8 Hz), 3.59 (3 H, m), 3.39 (1 H, dd, *J* = 10.3, 5.1 Hz), 2.43 (3 H, s); ^13^C NMR (DMSO-*d_6_*, 150 MHz) *δ* 195.5, 159.1, 138.6, 137.8, 135.1, 132.4, 131.8, 129.6, 128.6, 124.9, 117.2, 114.6, 71.7, 71.2, 70.2, 69.0, 63.3; HRMS (ESI) calcd. for C_19_H_21_N_2_O_6_: 373.1400; found: *m/z* 373.1394 (M^+^ + H).

*3-((2S,3R,4R,5S,6R)-3,4-dihydroxy-6-(hydroxymethyl)-5-((2S,3R,4S,5S,6R)-3,4,5-trihydroxy-6-(hydroxymethyl)tetrahydro-2H-pyran-2-yloxy)tetrahydro-2H-pyran-2-yloxy)-1-(1H-naphtho[2,3-d]imidazol-2-yl)pentane-1,2,4,5-tetraol* (**12**): yellow solid; mp = 180–183 °C; ^1^H-NMR (DMSO-*d_6_*, 600 MHz) *δ* 8.08 (s, 1H), 7.98 (d, *J* = 8.1 Hz, 2H), 7.91 (s, 1H), 7.36 (m, 2H), 5.51 (1 H, d, *J* = 6.2 Hz), 5.13 (1 H, s), 4.8 (1 H, d, *J* = 5.9 Hz), 4.22 (1 H, dd, *J* = 9.3, 4.6 Hz), 3.79-3.25 (16 H, m); ^13^C-NMR (DMSO-*d_6_*, 150 MHz) *δ* 161.4, 144.1, 135.4, 130.2, 129.8, 128.5, 127.9, 124.0, 123.3, 115.0, 107.2, 101.3, 100.7, 81.6, 80.1, 74.0, 73.8, 73.7, 73.1, 73.0, 72.4, 71.9, 70.4, 69.7, 63.2, 61.3, 60.6, 60.2; HRMS (ESI) calcd. for C_28_H_3__9_N_2_O_15_: 643.2350; found: *m/z* 643.2352 (M^+^ + H).

*3-((2S,3R,4R,5S,6R)-3,4-dihydroxy-6-(hydroxymethyl)-5-((2S,3R,4S,5S,6R)-3,4,5-trihydroxy-6-(hydroxymethyl)tetrahydro-2H-pyran-2-yloxy)tetrahydro-2H-pyran-2-yloxy)-1-(7H-purin-8-yl)pentane-1,2,4,5-tetraol* (**21**): yellow solid; mp = 163−164 °C; ^1^H-NMR (DMSO-*d_6_*, 600 MHz) *δ* 7.91 (1 H, s), 7.63 (1 H, s), 5.06 (1 H, s), 5.01 (1 H, d, *J* = 2.8 Hz), 4.38 (1 H, t, *J* = 7.9 Hz), 3.68-3.06 (17 H, m); ^13^C-NMR (DMSO-*d_6_*, 150 MHz) *δ* 154.3, 148.9, 135.6, 126.2, 109.0, 101.1, 100.8, 85.0, 80.0, 79.7, 77.4, 75.9, 73.8, 73.6, 73.5, 72.8, 72.3, 72.0, 70.2, 61.1, 60.8, 60.6; HRMS (ESI) calcd. for C_2__2_H_3__5_N_4_O_15_: 595.2099; found: *m/z* 595.2091 (M^+^ + H). 

*3-((2S,3R,4R,5S,6R)-3,4-dihydroxy-6-(hydroxymethyl)-5-((2S,3R,4S,5S,6R)-3,4,5-trihydroxy-6-(hydroxymethyl)tetrahydro-2H-pyran-2-yloxy)tetrahydro-2H-pyran-2-yloxy)-1-(1H-imidazo[4,5-c]pyridin-2-yl)pentane-1,2,4,5-tetraol* (**22**): yellow solid; mp = 102–104 °C; ^1^H-NMR (DMSO-*d_6_*, 600 MHz) *δ* 7.74 (1 H, s), 7.61 (1 H, d, *J* = 5.0 Hz), 6.47 (1 H, d, *J* = 5.0 Hz), 5.23 (1 H, d, *J* = 7.3 Hz), 5.07 (1 H, d, *J* = 2.5 Hz), 5.02 (1 H, d, *J* = 2.8 Hz), 4.34 (1 H, t, *J* = 8.1 Hz), 3.70-3.06 (17 H, m). ^13^C-NMR (DMSO-*d_6_*, 150 MHz) *δ* 144.0, 141.3, 139.4, 134.9, 129.5, 108.7, 101.3, 101.0, 80.4, 80.0, 77.6, 76.1, 74.0, 73.8, 73.7, 73.2, 73.0, 72.5, 72.2, 70.4, 61.3, 61.0, 60.8. HRMS (ESI) calcd. for C_23_H_3__6_N_3_O_15_: 594.2146; found: *m/z* 594.2153 (M^+^ + H).

*3-((2S,3R,4R,5S,6R)-3,4-dihydroxy-6-(hydroxymethyl)-5-((2S,3R,4S,5S,6R)-3,4,5-trihydroxy-6-(hydroxymethyl)tetrahydro-2H-pyran-2-yloxy)tetrahydro-2H-pyran-2-yloxy)-1-(4H-imidazo[4,5-c][1,2,5]oxadiazol-5-yl)pentane-1,2,4,5-tetraol* (**23**): white solid; mp = 118–121 °C; ^1^H-NMR (DMSO-*d_6_*, 600 MHz) *δ* 7.91 (1 H, s), 7.63 (1 H, s), 5.06 (1 H, s), 5.01 (1 H, d, *J* = 2.8 Hz), 4.38 (1 H, t, *J* = 7.9 Hz), 3.68-3.06 (17 H, m); ^13^C-NMR (DMSO-*d_6_*, 150 MHz) *δ* 149.4 (2 ×), 111.0, 100.8, 100.7, 80.3, 79.7, 76.6, 75.2, 73.7, 73.5, 73.4, 73.0, 72.7, 72.1, 71.9, 70.1, 61.0, 60.9, 60.5; HRMS (ESI) calcd. for C_20_H_3__3_N_4_O_16_: 585.1892; found: *m/z* 585.1884 (M^+^ + H).

## Conclusions 

We have achieved a simple, efficient and environmentally friendly process for saccharide labeling using iodine as a catalyst. In the presence of this reagent various aldoses react readily with aromatic diamines in acetic acid solution to form the corresponding aldo-imidazoles. The yields are generally excellent, without byproducts, and the reaction times are also short under mild and ambient conditions. In contrast to the parent saccharides, the aldo-imidazoles have a chromophore and display enhanced signals in MALDI–TOF MS analysis. This is potentially useful for the analysis of saccharides. We have demonstrated an application in carbohydrate compositional analysis via the aldo–imidazole derivatives by using capillary electrophoresis. Here, we also investigated the ionization efficiency of these aldo-imidazoles by using MALDI–TOF MS for saccharides detection. The aldo–imidazole derivatives might be further applied to other novel saccharides. This is a first example of the use of iodine as an oxidative agent in aldo–naphthylimidazole synthesis for saccharide labeling.

## Figures and Tables

*X-ray crystallographic data:* The CCDC deposition number of compound **1** is CCDC 768210. This data can be obtained free of charge *via* www.ccdc.cam.ac.uk/conts/retrieving.html.
